# Musculoskeletal health, work-related risk factors and preventive measures in hairdressing: a scoping review

**DOI:** 10.1186/s12995-019-0244-y

**Published:** 2019-08-17

**Authors:** Agnessa Kozak, Tanja Wirth, Miet Verhamme, Albert Nienhaus

**Affiliations:** 1 Competence Centre for Epidemiology and Health Services Research for Healthcare Professionals (CVcare), Institute for Health Services Research in Dermatology and Nursing (IVDP), University Medical Centre Hamburg-Eppendorf, Hamburg, Germany; 2 Unie van Belgische Kappers vzw, Ghent, Belgium; 3 Department of Occupational Medicine, Hazardous Substances and Public Health, Institution for Statutory Accident Insurance and Prevention in the Health and Welfare Services (BGW), Hamburg, Germany

**Keywords:** Musculoskeletal disorders, Hairdressers, Work-related risk factors, Prevalence, Prevention, Scoping review

## Abstract

**Background:**

Hairdressers are exposed to various work-related biomechanical and organizational risk factors. To date, there has been no overview of the evidence for this occupational group. The purpose of this scoping review is to gain insight into the current state of research on work-related musculoskeletal disorders (MSD) in hairdressing.

**Methods:**

Studies published up to November 2018 were identified by a systematic search using electronic databases (MEDLINE, PUBMED, CINAHL, Web of Science, LIVIVO), Google Scholar and reference lists of articles. Studies were screened by two researchers and synthesized in a descriptive manner.

**Results:**

Overall 44 studies with different study designs, scopes and approaches were included. Nineteen studies provided data on MSD prevalence in at least one body site. The prevalence values between the studies varied considerably. On average, the highest 12-month prevalence was reported for the lower back (range 13–76%), neck (range 9–58%), shoulder (range 28–60%) and hand/wrist (range 11–53%). In comparison to other occupational groups, hairdressers reported more frequent MSD in all body regions or exhibited a greater risk of leaving the profession for health reasons. Common risk factors include working with arms above shoulder level, repetitive movements, forceful exertion of upper extremities, awkward back postures and movements, high mechanical workload and standing. In addition to physical stress, lack of adequate breaks, overtime, and general distress may deteriorate health and performance of hairdressers. Three rehabilitative and three preventive interventional studies were found. Only the rehabilitative studies showed positive effects on the management of physical and mental strain and resulted in significant pain reduction, increased physical capacity and knowledge of potential risk factors for MSD.

**Conclusion:**

This data provides some evidence for work-related risk factors for MSD in hairdressers and indicate that there should be an intense focus on preventive measures. High quality and long-term interventional studies are needed to clarify the effectiveness of complex preventive concepts in hairdressing.

**Electronic supplementary material:**

The online version of this article (10.1186/s12995-019-0244-y) contains supplementary material, which is available to authorized users.

## Background

Musculoskeletal disorders (MSD) are common in the working age population and are conditions that affect passive (bones, joints) and/or active structures of the body (muscles, tendons, ligaments, peripheral nerves) [1]. Since MSD account for a high proportion of compensable occupational diseases worldwide many efforts have been undertaken to ascertain the potential risk factors in the development of MSD and its prevention in the workplace setting [2]. MSD are highly prevalent in manual-intensive occupations such as manufacturing, construction or services [3–5]. Hairdressers are a group of workers whose working ability and health condition may be affected by specific work-related activities. A daily task analysis showed that experienced hairdressers spend on average 29% of their time cutting, 17% dying, 10% blow-drying and 8% washing hair. These activities required frequent sagittal or lateral bending and twisting of the back (e.g. washing hair at the sink), static postures and long-standing periods. Repetitive tasks have been observed during all client-related activities [6]. Results from kinematic posture analysis revealed that hairdressers spend 9–13% of their total working time with arms elevated over 60° [7, 8]. Working with elevated arms above shoulder level is considered a major risk factor for clinically verified shoulder disorders or persistent severe pain [9, 10]. The relatively high force exertion and wrist velocity – combined with prolonged exposure – may account for the higher rate of hand/wrist pain, especially in female hairdressers [11]. In a study on the working conditions of Finnish hairdressers, the most hazardous factors for health were repetitive movements, awkward working postures, standing, uncomfortable temperatures and chemicals. The same factors – in addition to mental stress – were reported to cause work-related illnesses [12]. According to three health insurance companies with 51,842 hairdressers in Germany, MSD was the main reason for sick leave, within the range of 16 to 21% of the total [6]. Compared to other occupational groups, hairdressers complain significantly more frequently about MSD in different body regions [13, 14]. Studies indicate that hairdressers give up their profession mainly for health reasons. The most frequently cited reasons are complaints of the musculoskeletal system [15–17].

To understand the impact of working conditions on MSDs among this occupational group requires quantification of the MSD frequency, disability or injury, the identification of potential risk factors for these health consequences as well as effective preventive or rehabilitative measures. This is the first attempt to systematically map the current state of research on these aspects by synthesizing empirical, measurement-based or interventional studies in hairdressing.

## Methods

Due to a variety of study designs and a lack of summary of evidence we decided to conduct a scoping review. The general purpose of a scoping review is to examine the extent and nature of research activity, summarize the relevant research findings and to identify research gaps. Often a scoping review is conducted to determine whether a full systematic review should be undertaken and to guide future research [18]. In contrast to a systematic review the questions are answered in a descriptive way without critically appraising the study quality [19, 20]. For methodological purposes, we implemented the framework for a scoping review as adopted by Arksey and O’Malley [18]. The five stages have been implemented as follows:

### Stage 1: identification of the research question

In contrast to a systematic review the research question of a scoping review is broader and may include a large body of studies with various study designs and approaches [18]. Thus, the following question should be answered:

*“What is known from the existing literature about the frequency of MSD, work-related risk factors and measures to prevent or reduce MSD in hairdressers?”*

Consequently, we were seeking to present an overview of all thematically relevant material in a clear and comprehensible manner. Therefore, the study results were summarized and analyzed by applying a thematic approach based on the three subsections of the study question:

1. What is the prevalence and/or incidence of MSD in the different body regions?
2. What work-related risk factors are associated with MSD and what work activities are potential MSD hazards?
3. What work-related measures are applied to prevent or reduce MSD in hairdressers?

Through the iterative process of charting and analyzing the data in scoping reviews, further relevant result categories related to MSD in hairdressers were identified and presented as follows [18]:

- health reasons for leaving the hairdressing trade;
- self-reported strategies and barriers to reducing or preventing MSD.

### Stage 2: identifying relevant studies

A systematic literature search was conducted in the electronic databases MEDLINE, PUBMED, CINAHL, Web of Science and LIVIVO. The search string was first compiled for PUBMED and then adapted to other databases. The literature search contained terms for population and outcome. The detailed search strategy and database search can be obtained from the Additional file 1: Tables S1-B and S1-C. We also searched the reference lists of identified articles. In addition, we searched Google Scholar by using the terms hairdresser AND musculoskeletal disorder. The search included peer- and non-peer reviewed literature published from the inception of each database up to August 17, 2017 (last update Nov 5, 2018).

### Stage 3: study selection

Studies on musculoskeletal health were considered for the analysis if they reported separate results for hairdressers, assessed MSD prevalence or incidence, confirmed diagnosis (e.g. carpal tunnel syndrome), work-related risk factors and preventive or rehabilitative measures against MSD. Since the scoping review not only focused on clinical outcomes, biomechanical studies were also considered. The results of these studies are summarized under the second research question. The following inclusion and exclusion criteria were applied:

(i) *Population:* includes hairdressers who continue to work in their job and those who have changed or left their profession for health reasons. Also other related professions such as cosmetologists were considered;
(ii) *Exposure*: includes ergonomic, biomechanical, organizational and psychosocial factors which occur in the occupational context of hairdressers;
(iii) *Intervention*: includes all interventions that aim to prevent or reduce work-related MSD;
(iv) *Outcome*: includes health disorders related to musculoskeletal system such as (recurrent) pain, discomfort, tingling, numbness, stiff joints, swelling or dull aches. In addition, when stated, medically confirmed diagnoses were considered;
(v) *Study design:* includes peer review and non-peer-review publications except editorials, commentaries, conference papers, case reports, policy statements or expert opinions.

Two reviewers (AK, TW) independently assessed the title, abstract and full text of the articles. In the event of disagreement, a consensus was achieved through discussion. Reports published in English, German, Dutch, French, Italian, Portuguese and Spanish were included. The reports in Dutch were translated; all other full texts were read by experienced colleagues and then discussed with the study authors. A detailed description of the eligibility criteria as well as the search strategy are listed as Additional file 1: Table S1-A.

### Stage 4: charting the data

Data from included studies were entered into a standardized data charting form using the Excel spreadsheet. General information on author(s), year of publication, study location, study design, publication type, study aim, participant characteristics, methodology and outcome measures were recorded. The relevant study results were extracted separately and later reported according to the three subsections of the study question. The data was extracted by one person (AK) and verified by another reviewer (TW).

### Stage 5: collating, summarizing and reporting the results

The results on disease measures and risk factors were summarized descriptively and presented in separate tables. Where indicated, 12-months and/or point prevalence data were extracted. Prevalence figures and 95% confidence intervals (CIs) were calculated from the available data using modified Wald method [21]. All potential work-related risk factors examined in the studies were extracted and grouped into superordinate risk categories. Where possible, risk estimates were calculated by the authors.

## Results

**Fig. 1:**
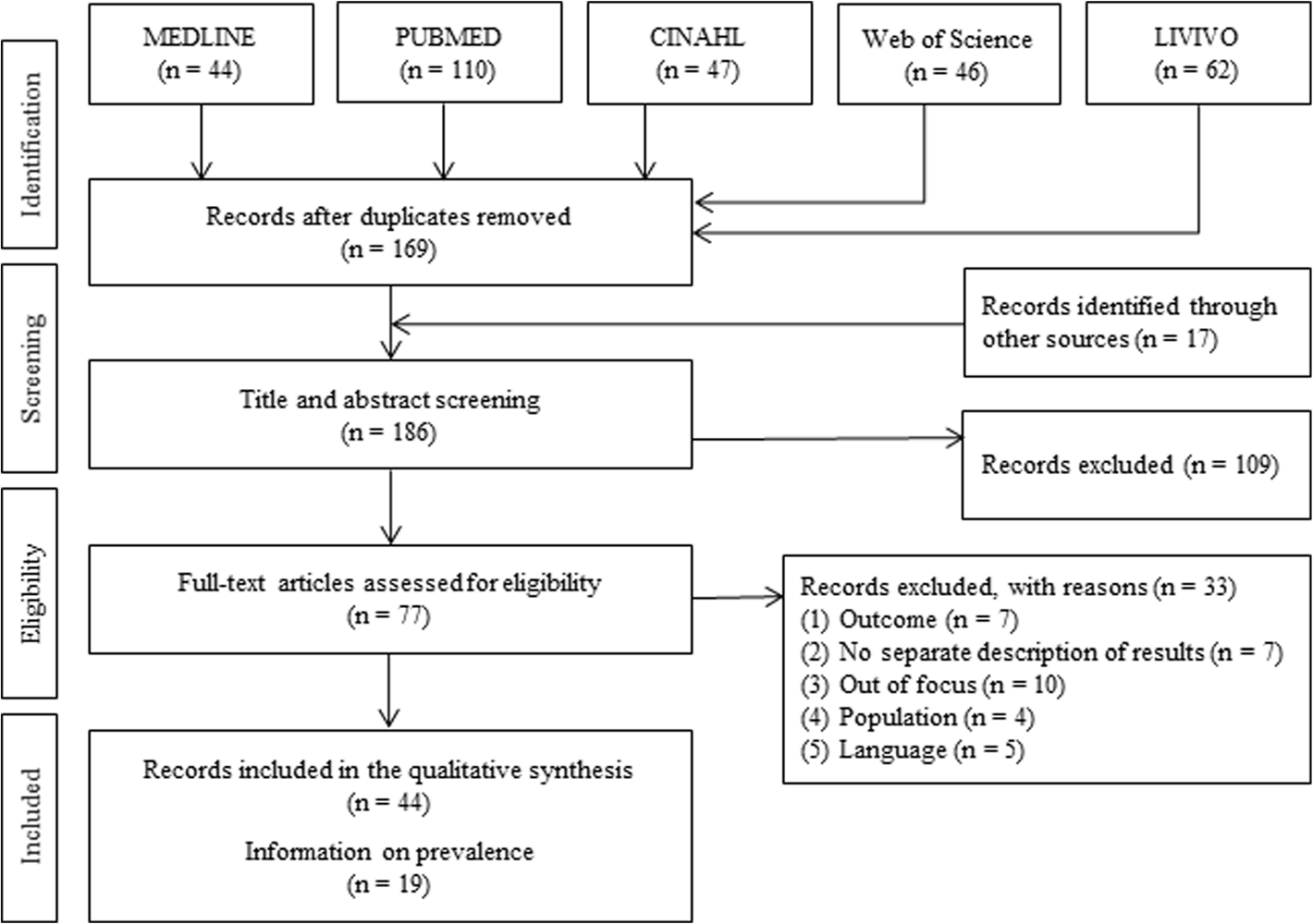
Flow diagram of the study selection

Our search strategy identified 186 articles, of which 44 met the eligibility criteria for the qualitative data synthesis (see Fig. 1). The characteristics of the included studies are listed as Additional file 2: Table S2. Of the eligible studies 29 were conducted in European countries. The majority (84%) of the included studies were published in peer-review journals. Most studies (89%) were published after the year 2000, which indicates that research in this occupational setting has recently increased. Of these, one study applied a qualitative study design with interviews [22] and three were national surveys of occupation-specific data which included hairdressers [23–25]. One study examined trends in compensation claims for work-related MSD [26]. Furthermore, seven studies were related to evaluation research [7, 27–32], three studies solely measured working postures while performing regular hairdressing tasks [8, 11, 33] and three studies were from the same cohort of students entering working life [34–36]. All but one study predominantly included females [37]. In one study, only cosmetologist were queried [38].

### Research question 1: prevalence and/or incidence of MSD in the different body regions

In total, 19 studies provided data on MSD prevalence in at least one body site and were presented depending on the given time frame, e.g. 12-month or point MSD prevalence [7, 13, 14, 25, 38–52]. If no time frame was explicitly stated, we judged it as point prevalence. Table 1 presents prevalence data for the spinal segments and Table 2 presents data on upper and lower extremities. The most frequently examined body regions were the lower back, neck, shoulder and hand/wrist. The prevalence values between the studies varied considerably. On average, the highest 12-month MSD prevalence was reported for the lower back (range 13–76%), neck (range 9–58%), shoulder (range 28–60%) and hand/wrist (range 11–53%). With some exceptions, the point prevalence was on average lower. The overall MSD prevalence (range 23–76%) with no specification of body site and time frame was on average above 50%.

**Table 1:** MSD prevalence of the spine segments

#	First author, year	Country	Sample size	Lower back		Neck		Upper back		Overall MSD^c^
Prevalence % (95% CI)^b^				point	12-month	point	12-month	point	12-month	12-month (†point)
1	Adewumi-Gunn, 2016 [39]	US	22	36% (19.6–57.1)	–	–	–	–	–	–
2	Amodeo, 2004 [40]	FR^d^	389	–	47% (41.6–51.5)	–	37% (31.9–41.4)	–	36% (31.4–40.9)	–
3	Aweto, 2015 [41]	NI	299	–	76% (71.1–80.7)	–	46% (40.6–51.8)	–	5% (2.7–7.8)	76% (70.4–80.1)
4	Bradshaw, 2011 [13]	UK^d^	147	42% (34.5–50.3)	–	31% (24.3–39.2)	–	27% (20.6–34.9)	–	–
5	Cruz, 2015 [42]	PO^d^	30	100% (88.4–100)	–	77% (58.8–88.5)	–	17% (6.9–34)	–	–
6	De Smet, 2009 [43]	BE^d^	145	–	–	–	–	–	–	41% (33.0–48.8)†
7	Deschamps, 2014 [44]	FR^d^	199	27% (20.9–33.2)	–	20% (14.7–25.7)	–	–	–	67% (60–73)†
8	Douwes, 2001 [45]	NL^d^	280	23% (18.3–28.1)	34% (28.3–39.3)	–	52% (46.3–57.9)	–	–	49% (42.8–54.4)
9	Hassan, 2015 [14]	EG	80	–	13% (6.7–21.7)	–	9% (4.0–17.2)	–	–	–
10	Mahdavi, 2013 [46]	IR	172	–	59% (51.3–65.8)	–	52% (44.9–59.7)	–	40% (32.5–47)	–
11	Mandiracioglu, 2009 [47]	TU	1284	–	27% (24.7–29.5)	–	–	–	–	32% (29.4–34.5)
12	Mussi, 2008 [48]	BR	220	–	39% (32.9–45.7)	–	47% (40.3–53.4)	–	–	71% (64.6–76.5)
13	O’Loughlin, 2010 [50]	AS	238	–	71% (65.4–76.8)	–	–	–	–	–
14	Omokhodion, 2009 [51]	NI	355	19% (15.1–23.3)	–	–	–	–	–	–
15	Puckree, 2009 [52]	SF	75	39% (28.4–50)	–	–	–	11% (5.3–19.9)	–	60% (48.7–70.4)†
16	Schneider, 2006 [25]	GE^d^	26	46% (28.8–64.6)	70% (49.9–83.7)	–	–	–	–	–
17	Tsigonia, 2009 [38]	GR^d^	102	–	53% (43.3–62.3)	–	58% (48.1–67)	–	–	–
18	Veiersted, 2008 [7]	NO^d^	188	–	–	28% (22.2–35)	47% (40.3–54.5)	–	–	–
Range (min-max in %)				19–100%	13–76%	20–77%	9–58%	11–27%	5–40%	32–76%
No of studies with data on prevalence				*n* = 16		*n* = 11		*n* = 6		*n* = 7

*Abbreviations*: *CI* confidence interval, *MSD* musculoskeletal disorders

^a^Only hairdressers

^b^95% confidence interval (CI) not presented in articles but calculated from sample size and prevalence estimate

^c^Overall MSD: prevalence without body area indication

^d^European countries

**Table 2:** MSD prevalence of the upper and lower extremities

#	First author, year	Country	Sample Size^a^	Shoulder		Hand/wrist		Finger	Elbow	Knee	Feet
	Prevalence % (95% CI)^b^			point	12-month	point	12-month	point (†12-months)	12-month (†point)	12-month	12-month (†point)
1	Adewumi-Gunn, 2016 [39]	US	22	–	–	54% (34.7–73.1)	–	54% (34.7–73.1)	–	–	–
2	Amodeo, 2004 [40]	FR^c^	389	–	28% (23.3–32.2)	–	19% (15.2–23)	–	4% (2.7–6.9)	–	–
3	Aweto, 2015 [41]	NI	299	–	60% (54.6–65.6)	–	25% (20.8–60.7)	27% (22.4–32.4)†	15% (11.4–19.6)	33% (27.7–38.3)	24% (19.3–28.9)
4	Bradshaw, 2011 [13]	UK^c^	147	37% (30–45.5)	–	29% (22.5–37.1)	–	–	7% (4.1–13)†	–	35% (27.5–42.7)†
5	Cruz, 2015 [42]	PO^c^	30	83% (66–93.1	–	43% (27.4–60.8)	–	–	–	–	–
6	Deschamps, 2014 [44]	FR^c^	199	28% (22.4–34.8)	–	10% (6.5–15.1)	–	9% (5.7–13.9)	8% (4.9–12.7)†	–	–
7	Douwes, 2001 [45]	NL^c^	280	–	48% (42.1–53.7)	–	26% (21.3–31.5)	–	7% (4.6–10.8)	–	–
8	Hassan, 2015 [14]	EG	80	–	13% (6.7–21.7)	–	11% (5.8–20.2)	–	14% (7.7–23.1)	4% (0.8–10.9)	10% (4.9–18.8)
9	Mahdavi, 2013 [46]	IR	172	–	49% (42–56.8)	–	49% (41.5–56.3)	–	14% (9.5–20)	41% (34.2–48.8)	20% (15–27)
10	Mussi, 2008 [48]	BR	220	–	49% (42.6–55.7)	–	–	–	–	–	–
11	O’Loughlin, 2010 [50]	AS	238	–	–	–	–	–	–	–	44% (38–50.5)
12	Nordander, 2013 [49]	SE^c^	78	–	–	33% (23.8–44.4)	49% (38–59.6)	–	–	–	–
13	Tsigonia, 2009 [38]	GR^c^	102	–	35% (26.7–45)	–	53% (43.3–62.3)	–	–	28% (20.6–37.9)	–
14	Veiersted, 2008 [7]	NO^c^	188	32% (26.2–39.4)	53% (45.5–59.7)	–	–	–	–	–	–
Range (min-max in %)				32–83%	28–60%	10–54%	11–53%	9–54%	4–15%	4–41%	10–44%
No of studies providing data on prevalence				*n* = 11		*n* = 11		*n* = 3	*n* = 7	*n* = 4	*n* = 5

*Abbreviations*: *CI* Confidence interval, *MSD* Musculoskeletal disorders

^a^Only hairdressers;

^b^95% confidence interval (CI) not presented in articles but calculated from sample size and prevalence estimate

^c^European countries;

A study from France examined trends in hairdressers’ compensation claims for the years 2010–2016. The overall claim rate for work-related MSD increased by 12.8% (n.s.). Permanent incapacity (incidence rate 2/1000) and the number of lost work days increased significantly by 16% during the study period. In total 666,461 days were lost due to work-related MSD [26].

### Comparisons to other professional groups regarding MSD frequency

A National German Health Survey provided a representative analysis of back pain prevalence by occupation category. Hairstylists/beauticians were among the top five professions with the above-average prevalence for back pain (e.g. the 1-year and 7-day prevalence were 69.7 and 47.3% respectively) [25]. According to the U.S. National Health Interview Survey on back pain, female hairdressers belong to the top 6 high-risk occupations for back pain [23]. Epidemiologic surveillance data on carpal tunnel syndrome (CTS) from Maine and Loire regions in France showed that a substantial proportion of new CTS cases (between 2002 and 2004) among female hairdressers were attributable to work (attributable risk fractions 86.6%). Thus, they belong to the top 10 high-risk occupations for CTS [24].

In a case-control study, which was conducted with hairdressers and non-hairdressing controls, hairdressers reported significantly higher levels of MSD, including shoulder (OR 11.6, 95% CI 2.4–55.4), hand/wrist (OR 2.8, 95% CI 1.1–7.6), upper back (OR 3.8, 95% CI 1.0–14.9) or lower back pain (OR 4.9, 95% CI 1.5–15.9) [13]. In a further comparative study with office workers female hairdressers reported pain in all body regions significantly more often (neck 36% vs. 8%, shoulders 39% vs. 10% or hand/wrists 41% vs. 4%) [14]. A study from France analyzed data from occupational health examination of self-employed and wage-earning hairdressers. The risk of musculoskeletal injuries was significantly higher among the self-employed (66.8% vs. 29.7%) [44]. In a case-control study from Turkey the frequency of CTS (confirmed by nerve conduction studies) in female hairdressers was slightly higher compared to unemployed female control group (RR 1.35, 95%CI 0.98–1.84). In addition, they showed significantly higher pain intensity and functional loss levels. Hairdresser who were diagnosed with CTS worked significantly longer in their profession than those hairdressers without CTS [53].

### Research question 2: work-related risk factors and potentially hazardous work activities

Fifteen studies examined potential risk factors for work-related MSD in hairdressers relying on self-rating or statistical estimation (see Table 3). They varied greatly in the types of risk factors, the methods applied and in the reporting of the findings. The reported risk factors were synthesized into the following six main categories:

1. Strenuous hand or arm postures and movements (e.g. arms above shoulder, repetition) [12, 14, 36, 38, 41, 42, 45, 52]
2. Awkward postures and movements of the spine (e.g. bending and twisting the back) [12, 14, 38, 41–43, 45, 48, 52]
3. Workload and biomechanical strain (e.g. mechanical workload, overtime, no breaks) [34, 35, 38, 41–43, 45]
4. Prolonged standing and sitting [12, 14, 38, 52]
5. Other factors (e.g. work experience, mental stress and burnout, gender or low support) [12, 37, 38, 41, 43, 48]
6. Specific hairdressing tasks (e.g. cutting, dying or styling hair) [46, 54]

**Table 3:** Risk factors for work-related MSD in hairdressers

Identified risk factors	Outcome	Comparison category	Statistical measure (%^a^; OR; RR; r; *p*-value)	Author (year)
(1) Awkward postures and movements of the spine				
- working in static postures	WRMSD	–	91%	Aweto et al. (2015) [41]
- bending or twisting back	WRMSD	–	28%	
- constantly twisting the spine	WRMSD	–	53%	Cruz et al. (2015) [42]
- bending the spine forward > 50% of the time	back pain	–	sig. Correlation (*p* < 0.001)	Puckree (2009) [52]
- awkward back postures (back is bent or twisted)	WRMSD	–	64%	Tsigonia et al. (2009) [38]
- working postures	WRMSD and diagnosis	–	81 and 5%	Leino et al. (1999) [12]
- uncomfortable postures (body, neck, shoulders)	WRMSD	yes vs. no	OR 2.8 (95%CI 1.4–5.5)^b^	Mussi et al. (2008) [48]
- working with spinal rotation	WRULD	yes vs. no	OR 2.1, *p* < 0.05 ^b^	DeSmet et al. (2009) [43]
- awkward back postures	back pain	yes vs. no	RR >10^c^	Hassan et al. (2015) [14]
- working in static postures 6–8 h/day	WRMSD	VDU vs. HD work	OR 1.6 (95%CI 1.1–2.2)^c^	Douwes et al. (2001) [45]
(2) Strenuous hand or arm postures and movements				
- repetition of a task	WRMSD	–	71%	Aweto et al. (2015) [41]
- repetitive movements	WRMSD and diagnosis	–	66 and 5%	Leino et al. (1999) [12]
- position of arms at or above shoulder level	back pain	–	sig. Correlation (*p* < 0.001)	Puckree (2009) [52]
- working with equipment above shoulder level	WRMSD	–	63%	Cruz et al. (2015) [42]
- strenuous shoulder movements	shoulder pain / hand/wrist pain	yes vs. no	OR 6.0 (95%CI 1.7–21.5)^b^ / OR 25.3 (95%CI 2.8–229.1)^b^	Tsigonia et al. (2009) [38]
- strenuous shoulder movements	neck pain / shoulder pain	yes vs. no	RR 2.4 (95%CI 1.4–4.1)^c^ / RR 3.5 (95% CI 2.0–6.0)^c^	Hassan et al. (2015) [14]
- working with elevated arms	shoulder pain (score)	% working time > 60° % working time > 60° > 5 s	RR 1.3 (95%CI 1.1–1.5)^b^ / RR 2.0 (95%CI 1.5–2.6)^b^	Hanvold et al. (2015) [36]
- working with hands above shoulder level 6–8 h/day	WRMSD	VDU vs. HD work	OR 8.4 (95%CI 4.1–15.8)^c^	Douwes et al. (2001) [45]
- frequent elbow movements 6–8 h/day	WRMSD		OR 2.4 (95%CI 1.7–3.3)^c^	
- extreme wrist extension/flexion 6–8 h/day	WRMSD		OR 2.6 (95%CI 1.4–4.8)^c^	
- frequent manual material handling	neck pain / hand/wrist pain	yes vs. no	RR 3.1 (95%CI 1.4–6.8)^c^ / RR 2.6 (95%CI 1.3–4.9)^c^	Hassan et al. (2015) [14]
- frequent manual material handling	neck pain / knee pain	yes vs. no	OR 12.6 (95%CI 2.1–75.5)^b^ / OR 6.4 (95%CI 1.9–21.4)^b^	Tsigonia et al. (2009) [38]
(3) Workload and biomechanical strain				
- stress and working overtime	WRMSD	–	83 and 97%	Cruz et al.(2015) [42]
- no adequate uninterrupted breaks between clients	WRMSD	–	30%	Douwes et al. (2001) [45]
- no adequate rest breaks	WRMSD	–	72.4%	Aweto et al. (2015) [41]
- large number of clients per day and working overtime	WRMSD	–	92 and 94%	
- working at physical limit	WRMSD	–	34%	
- large number of clients per day	WRULD	< 8 vs. 10–15 clients	OR 6.7, *p *< 0.01^b^	DeSmet et al. (2009) [43]
**-** excessive work	WRULD	low vs. very high	OR 6.1, *p* < 0.01^b^	
- high perceived exertion	knee pain	yes vs. no	OR 5.3 (95%CI 1.4–21)^b^	Tsigonia et al. (2009) [38]
- high job demands	hand/wrist pain	yes vs. no	OR 7.6 (95%CI 1.8–32.1)^b^	
- putting intense effort on hands	WRMSD	–	63%	Cruz et al.(2015) [42]
- high mechanical workload	neck and shoulder pain / workload levels	workload score (0–24) / HD & EL vs. media & design trainees	RR 1.01 (95%CI 1.00–1.02)^d, b^ RR 1.36 (95%CI 1.3–1.5)^d, b^	Hanvold et al. (2014) [35]
- high sustained muscle activity	shoulder pain (score)	muscle activity (0–100%)^e^	median 52% (range 24–91%) r 0.2, *p *< 0.001	Hanvold et al. (2015) [36]
(4) Prolonged standing or sitting				
- standing during work > 75% of the time	back pain	–	sig. Correlation (*p* < 0.01)	Puckree (2009) [52]
- prolonged standing	WRMSD and diagnosis	–	65 and 1%	Leino et al. (1999) [12]
- prolonged standing	feet/leg pain / knee pain	yes vs. no	RR 5.3 (95%CI 1.8–15.4)^c^ / RR 21.0 (95%CI 2.8–156.7)^c^	Hassan et al. (2015) [14]
- prolonged standing and sitting	hand/wrist pain	yes vs. no	OR 55.7 (95%CI 8.8–354.9)^b^	Tsigonia et al. (2009) [38]
(5) Other factors				
- > 15 years in the profession	WRMSD	< 5 vs. 15–45 years	OR 3.0 (95%CI 1.2–7.9)^b^	Mussi et al. (2008) [48]
- years of work experience	DASH score / NPDI score	–	r 0.7, /r 0.7, p < 0.001	Kaushik & Patra (2014) [37]
- lack of acknowledgment and uncomfortable postures	WRMSD	1–23 vs. 29–35 score	OR 3.5 (95%CI 1.5–8.3)^b^	Mussi et al. (2008) [48]
- mental stress	WRMSD and diagnosis	–	51 and 2%	Leino et al. (1999) [12]
- burnout	WRULD	low vs. very high	OR 8.6, p < 0.001^b^	DeSmet et al. (2009) [43]
- bordering ambient temperature (high)	WRULD	yes vs. no	OR 2.5, p < 0.05^b^	
- female gender	WRULD	female vs. male	OR 3.1, p < 0.05^b^	
- sudden movements	WRMSD	–	12%	Aweto et al. (2015) [41]
- low co-worker support	back pain/ hand/wrist pain	yes vs. no	OR 7.6 (95%CI 1.8–32.1)^b^ / OR 5.1 (95%CI 1.2–21.4)^b^	Tsigonia et al. (2009) [38]
(6) Hairdressing task as risk factor for MSD				
- hair styling	WRULD	REBA index ^f^ (% high & very high risk for MSD)	69%	Mahdavi et al. (2013) [46]
- hair dying	WRULD		66%	
- hair cutting	WRULD		64%	
- trimming face	WRULD		62%	
- doing make up	WRULD		53%	
**-** trimming eye brows	WRULD		49%	
**-** shampooing hair at least 50%/day	WRMSD	OCRA index^g^	index 5.0	Mastrominico et al. (2007) [54]
**-** cutting hair at least 50%/day	WRMSD		index 8.1	
- styling hair at least 50%/day	WRMSD		index 9.4	
**-** dying hair at least 50%/day	WRMSD		index 9.0	

*Abbreviations*: *DASH* Disability of Arm, Shoulder, Hand Index, *EL* Electrician, *HD* hairdressers, *NPDI* Neck Pain Disability Index, *OCRA* Occupational Repetitive Action check list, *OR* odds ratio*, REBA* Rapid Entire Body Assessment, *RR* relative risk*, VDU* Visual Display Unit, *WRMSD* work-related musculoskeletal disorders/discomfort, *WRULD* work-related upper limb disorders/discomfort

^**a**^Self-rated risk factors for WRMSD/WRULD

^b^Results from adjusted analysis

^c^Data were calculated from the authors of the study

^d^Each increase in mechanical workload was associated with 1% increase in neck and shoulder pain in women (the majority in the group were female hairdresser (*n* = 163) compared to 5 female electrician trainees)

^e^Relative time of sustained trapezius muscle activity during the working day: low (0–29%), moderate (30–49%) and high (50–100%)

^f^REBA index: lower risk for MSD (< 3), moderate risk (4–7), high risk (8–10), very high risk (11–15)

^g^OCRA index: no risk for MSD (< 4.5), moderate risk (4.6–9), high risk (> 9)

Mastrominico et al. [54] showed that all principal hairdressing activities performed for at least 50% of the working day exhibited intermediate to high risk for work-related upper limb disorders (WRULD). Similarly, Mahdavi et al. [46] found that 61% of studied postures could be classified as high risk postures for work-related MSD.

The following studies examined hairdressing activities and/or the corresponding body postures and movements. In a study by Chen et al. [11], the mechanical exposure of hairdresser’s and barber’s wrists were assessed by using electromyography (EMG). Female hairdressers exhibited significantly greater EMG activity (*p* < 0.001) and faster extensions-flexion speed (velocity) in their non-dominant hand (*p* < 0.001) than their male counterparts. The authors concluded that high force exertion and wrist velocity combined with prolonged exposure may account for the greater rate of hand/wrist pain in female hairdressers.

Wahlström et al. [8] analyzed upper arm postures and movements in female hairdressers by using inclinometers. They found that the exposure for the left and right hand was similar. On average, hairdressers spent 6.8% (33 min) of their working time with their right arm abducted at > 60°; for the left arm it was 5.5% of the total working day. Similar results were found by Veiersted et al. [7]. In a pilot study from Portugal, 77% of the hairdressers reported that they performed their activities in a standing position, 17% in a sitting position with rotation of the spine and 7% in a sitting position with elevated arms above shoulder level. In regards to upper limb activities during work, 30% performed repetitive and dynamic movements and 60% elevated objects above shoulder level (> 60°) [42]. Figueiredo da Rocha and Simonelli [55] found that hair straightening with a round brush requires high mechanical overload of the cervical and spinal columns (e.g. straightening curly hair takes up to 1 hour). Moreover, the upper limbs are strained from repetitive movements in protracted extended positions. They concluded that the daily workload of hairdressers is high and aggravated by the lack of regular breaks. Similar results were found in a Dutch study. More than 6 hours of repeatedly using the wrist and elbow as well as working in static positions caused the greatest strain on the musculoskeletal system. These movements are predominantly triggered by tasks such as blow drying and cutting, which comprised up to 82% of the working day. The lack of sufficient uninterrupted breaks contributed to the strain experienced by hairdressers [45].

The previous results are also supported by an objective job tasks analysis. During the working day, hairdressers often abducted their upper arms on both sides, which was combined with static holding phase (> 4 s). Moreover, they often had to stretch their arms over the shoulder level and perform tasks with horizontal adduction of the arms. While washing and cutting hair, hairdressers often had to bend forward or twist their spines and work in prolonged static postures. This poor posture was often combined with hunched back. Those who used the rolling stool often exhibited a steeply inclined lumbar spine and had to raise their hands more often above shoulder level [6]. The same authors report that during cutting, dying and blow-drying, more than 25% of time was spent in flexion (angles > 20° and > 60°) and abduction (> − 20° and > − 60°) for both shoulders. Pronation (> 20 and > 40°) of both elbows was observed during all tasks. Extension (> − 25° and > − 50°) of the left hand was observed for cutting and washing hair. A high proportion of time with forward curvature of the spine was recorded during cutting (66%), washing (62%) and dying (36%). All four hairdressing tasks led to highly repetitive actions of the upper extremities. The Kilbom reference values [56] for high repetition in the shoulder (high risk for MSD: > 2.5 rep/min), and for the elbow and hand (> 10 rep/min) were both significantly exceeded, particularly when using the round brush to straighten hair (e.g. right hand 50 rep/min) [33].

### Comparisons to other professional groups regarding work-related risk factors

A prospective study from Norway followed a young cohort of students from technical schools entering working life. After 2.5 years of follow-up, hairdresser students exhibited the greatest median pain in the neck-shoulder region, as compared to the other students. Concurrently with these results, hairdressers had the highest median sustained muscle activity of 52% of the total working day in contrast to electricians (33%), various jobs (27%) and other students (10%). The relative time of sustained muscle activity showed a significant correlation with pain (r = 0.21, *p* < 0.001) [34]. When compared to other female students, hairdressers spent longer working times with arms elevated at > 30° (45% vs. 35%), > 60° (11% vs. 1%) and > 90° (2% vs. 0.4%). For every additional unit increase in arm elevation of more than 60°, an estimated 28% increase in shoulder pain was found among female students [36]. Moreover, the authors observed a significant increase in the prevalence of moderate/severe pain for female students (majority hairdressers) over the course of 6.5 years (RR 1.5, 95% CI 1.24–1.81). Mechanical workload and perceived muscle tension were identified as risk factors for neck and shoulder pain in women [35].

Nordander et al. [49] explored the exposure-response relationship between work-related risk factors and MSD in elbows and hands. The mean value for palmar wrist flexion, expressed as the 90th percentile, was greater for hairdressers than the overall mean for other occupations (21° vs. 10°). With respect to static and peak load of muscular activity, expressed as the 10th or 90th percentile of maximal voluntary contraction (% MVC), hairdressers showed higher static (4.5% vs. 1.8%) and peak loads (35% vs. 26%) of the right-hand muscles [49].

### Research question 3: work-related preventive and rehabilitative measures

Seven studies addressed evaluation research. Three studies described preventive and three rehabilitative measures. One study evaluated new Ergonomic Tool Design (ETD) scissors.

#### Preventive work-related measures

Bertozzi et al. [29] assessed the effect of an exercise program targeted to the cervical and lumbar spine in combination with an ergonomic brochure. The control group received only the brochure. After 6 weeks of intervention no significant differences were found in pain intensity or level of disability between the exercise and control groups. Similarly, Veiersted et al. [7] examined the effect of a short-term intervention, including five recommendations on working techniques to reduce neck and shoulder workload, such as working with less elevated arms and relaxing the upper body and follow-up instructions. The control group received a brochure with corresponding illustrations. Time spent with highly elevated upper arm postures above 90° was reduced from 4 to 2.5%. No intervention effect was detected on muscular load, velocity of arm movements or neck and shoulder complaints.

In a further study by Crippa et al. [31], young trainees were provided with an education program on the prevention of risks related to skin, respiratory or upper limb disorders. At the beginning of the school training and 2 years later their knowledge of risks, work-related symptoms and adopted preventive measures was assessed. Positive effects on their knowledge preventive measures and work-related dermatitis were observed. However, the rates for lower back pain (9 to 36%) and shoulder or elbow pain (3 to 15%) increased significantly over the training years.

#### Rehabilitative work-related measures

Three studies from Finland evaluated the effectiveness of occupationally oriented medical rehabilitation courses on changes in working techniques, subjective well-being, physical and muscular capacity, MSD, perceived work ability or redesign of workplaces [27, 28, 32]. The courses were addressed to hairdressers and other occupations with a history of chronic neck-shoulder or back pain. In the studies from Arokoski et al. [27, 28], hairdressers reported significant reductions in subjective physical and mental strain, neck-shoulder and back pain as well as visits to the doctor due to MSD after the rehabilitation [27, 28]. When asked for subjective reasons for the decrease in strain the following aspects were mentioned: use of new working techniques, frequent use of a chair, use of exercise breaks, increased physical fitness and new ability to relax during work [27]. In a similar study by Nevala-Puranen et al. [32], hairdressers with a history of MSD underwent a rehabilitation course that addressed workplace redesign, theoretical knowledge, physical exercises, and discussion of interpersonal relations or stress. In addition, habitual work techniques were videotaped in simulated work situations. The video data was utilized in teaching ergonomics. For example, ergonomic techniques during hair cutting focused on using a chair, keeping arms near the body, and cutting with the wrists in a neutral position, relaxing the shoulders and asking customers to turn or bend the head. The new work techniques led to decreased activity of the right trapezius muscles from 6–12% to 3–8% MVC. Static, dynamic and peak muscle load decreased from 2 to 1%; 6–3% and 13–9% MVC, respectively. Correspondingly, the overall pain intensity decreased from 5.0 to 2.6 points on a visual analogue scale.

#### Evaluation of ergonomic tool design (ETD) in hairdressing

Boyles et al. [30] investigated the use of ETD scissors with a bend in the handles of 90°. In contrast to standard scissors, the ETD scissors allow the hand/arm to remain in a neutral position and below the shoulder level when cutting hair from any angle. In comparison to standard scissors, perceived pain scores (1–7) were significantly less for hand/wrist (2.1 vs. 1.3) and back/shoulder (2.0 vs. 1.4). The time spent in neutral position of the wrist increased (27.7% vs. 72.6%) and working with hand above shoulder level decreased (53.2% vs. 17.2%). Although initial use of ETD scissors was very unaccustomed, participants felt comfortable after some time.

### Other relevant aspects related to MSD in hairdressers

#### Health reasons for leaving the hairdressing trade

A Finnish study assessed the risk of leaving the profession for health and other reasons among female hairdressers as compared to workers engaged in commercial work. The relative risk of leaving the profession among hairdressers was increased by RR 2.7 (95% CI 1.1–6.3) for a repetitive strain injury of the wrist and elbow and by RR 1.7 (95% CI 1.2–2.5) for diseases of the neck or shoulders [16]. Two studies from Denmark examined the health reasons for leaving the hairdressing trade: one with retrospective and one with prospective study design. Among all former hairdressers the primary health complaint causing them to leave their job was musculoskeletal pain (42%) followed by hand eczema (23%), other diseases (21%) and allergy (18%) [17]. The prospective study showed that during the 3-year follow-up, 21.8% of the hairdressing apprentices had left the trade; of them 70.4% due to health complaints. The most frequently reported reasons were musculoskeletal pain (47.4%), followed by skin diseases (42.1%) and respiratory symptoms (23.7%) [15].

#### Strategies and barriers to reducing or preventing MSD in hairdressers

In a qualitative study with 14 female hairdressers, musculoskeletal stress was mentioned as one of several work-related symptoms. To provide some relief minor individual changes in work-techniques and use of products or physical training were employed. However, the hairdressers often failed to take further steps due to lack of knowledge or due to the financial restriction and organizational situation of the salon. At the beginning of their career, hairdressers put more effort into training and application of acquired skills; preventive work techniques were of secondary importance. The practice of good work routines depended on factors such as colleagues, personal knowledge or existing symptoms. Hairdressers’ awareness of the preventive work gained in importance when they started a business of their own [22]. In a study by Aweto et al. [41] more than half of the subjects reported the gradual onset of symptoms in the first 5 years of being a hairdresser. When asked which coping strategies they adopted to reduce MSD symptoms the hairdressers most often mentioned taking sufficient breaks (35.3%), not attending customers if this causes/worsens discomfort (18.5%) and modifying the working position (14.3%). The hairdressers also reported that the symptoms affected their daily activities, and thus their job efficiency. Some reported that the working activities aggravated an already existing injury (14.4%). According to Bradshaw et al. [13] more than half the hairdressers reported that they continued to work while suffering health problems as they are not able to take time off from work (36%), had a manageable disease (30%) or because they are self-employed (21%).

## Discussion

This is the first scoping review which provides an overview of the frequency of MSD, potential risk factors, preventive and rehabilitative measures and ergonomic findings in hairdressers. The most affected body sites are the back, neck, shoulder and wrist/hand. Physical strains are mainly caused by prolonged non-neutral postures along with forward flexion and backward extension of the trunk and repetitive movements of the upper extremities. Additional factors are lack of adequate breaks during work, working at high pace, general distress or prolonged standing periods. Several studies point out that MSD can occur in the first years at work [31, 35, 41]. This underlines the necessity and importance of early preventive measures in hairdressing. However, the available publications only provide relatively sparse evidence for effective preventive or rehabilitative actions. Studies on measures to prevent MSD have demonstrated virtually no reduction in pain or stress [7, 29, 31]. Possible reasons for the lack of evidence may be the following factors: uncontrolled study design, small study samples or short follow-up periods. Hairdressers who have suffered MSD of the back, neck or shoulder and who have already received rehabilitation treatment apparently benefit from newly learned ergonomic working techniques and newly purchased equipment [27, 28, 32]. The components of the rehabilitation programs may provide helpful approaches for the prevention of MSD. However, they are more extensive, prolonged and expensive than the preventive measures. At this point we should consider two typical and common activities performed by hairdressers that are classified in the publications as being stressful.

### Potentially harmful task: styling and blow-drying hair

The first is styling and drying hair with a circular brush – for which very high values for repetition have been measured that exceed thresholds [33, 45]. Continuous grasping the brush and hairdryer, in combination with physical postures and movements that may be extreme and non-ergonomic (e.g. shoulder abduction > 60°), require high peak loads and static stress on the muscles [8, 33, 54]. Mechanical stress, subjective muscular tension and working at shoulder height have been identified as risk factors for pain in the shoulders and neck in female apprentices in technical occupations [35, 36]. This observation has been confirmed by a recently published meta-analysis. The authors found moderate evidence for an association between physical stress and shoulder diseases for hand-arm elevation (OR 1.9, 95% CI 1.5–2.5), shoulder load (OR 2.0, 95% CI 1.9–2.1), as well as slight evidence for hand force exertion (OR 1.5, 95% CI 1.3–1.9) [57]. Older reviews also confirm these associations [58, 59]. The combination of repetition and low force exertion typically leads to a moderate increase in the risk of MSD. With high force exertion, the risk is greatly increased [60]. These risk factors are associated with the carpal tunnel syndrome [61] and other specific diseases of the elbow [62].

### Potentially harmful task: cutting hair

Much of the working day is taken up with cutting hair and this activity is also associated with risk. During this procedure, the wrist is permanently held in a non-neutral position (flexion and extension) while the scissors and comb are grasped precisely [11]. It has been shown that a large proportion of the time is spent with the left hand extended [11, 33]. Not only are the upper extremities stressed but also the upper and lower segments of the spinal column. One important malposition is the anterior curvature of the spinal column. Posterior extension of the cervical spine is also fairly common. In comparison to other activities, cutting hair involves relatively long periods (> 4 s.) with static curvature of the trunk and anterior or posterior inclination [33]. Usage of stools enhances abnormal straightening of the lumbar spine and can lead to additional structural stress. In addition, hairdressers who work when seated lift their arms higher than when working in the standing position [6].

### Potentially harmful aspect of work organization: lack of breaks

Another important factor is the possibility of taking a break between the stressful activities as this can prevent or alleviate micro injuries [60]. However, the available studies show that the physical loads during normal hairdressing work exceed tolerance thresholds and that regular breaks are rarely respected [31, 41, 43, 45]. The probability of tissue damage increases with the frequency and duration of biomechanical exposure [63].

### Strengths and limitations

In this scoping review we followed a systematic approach suggested by the framework of Arksey and O’Malley [18]. We included peer-review and non-peer-review literature published in several languages with no restriction to publication date. Thus, a wide range of literature formed the basis for this review. The general purpose of this scoping review was to examine the extent, range and nature of research activity. This study design is suitable for gaining an initial overview of scientific activities on the subject of MSD in hairdressers and for identifying research gaps. The validity of this review depends on the validity of the studies included. However, a major limitation to this scoping review is the lack of quality assessment of the individual studies. We did not assess the study quality, as there is no suitable instrument that could guarantee the comparability of the study concepts. When examining the studies, we noticed a number of shortcomings in reporting and in internal and external validity (e.g., insufficient description of the study population and inclusion criteria, little control of confounders or lack of objective assessment of exposure). Very different measurement instruments were used to assess the prevalence of MSD and biomechanical exposure. The comparability of the results must therefore be regarded as limited. At this point it is difficult to make general statements about the severity of the physical complaints and strains in this occupational group. We therefore consider that at present it would not be advisable to perform a systematic review.

## Conclusion

The presented data indicates that MSD frequently occurs, and that activities such as styling and cutting hair may contribute to the risk of hairdressers developing MSD. In comparison to other occupational groups hairdressers are frequently exposed to manual and static physical stress. In addition, there is a greater chance that hairdressers will leave their profession earlier in their career. Hence, this occupational group could benefit from preventive structural, operational and educational measures. However, only a limited number of intervention studies with inconclusive results are available that could provide some options for reliable action. Therefore, further comparative studies with high methodological quality are needed to evaluate multi-level strategies for the prevention of MSD in hairdressers, combining behavioral and organizational measures. The few studies from rehabilitation research indicate that behavioral measures such as instruction in healthy ergonomic procedures and physical exercises may improve physical fitness, muscle strength and ergonomic working techniques. Also it needs to be investigated whether measures at an organizational level, such as maintaining regular breaks, less overtime, mutual psychosocial support and the provision of ergonomic saloon equipment, may have an impact on hairdressers’ health and job satisfaction. If these measures are introduced early in training or in self-employment, they could probably lead to a sustainable decrease in occupational risks and improved health. To investigate the effect of individual and organizational measures on MSD in hairdressers, prospective controlled interventional studies are needed. Furthermore, the prevalence and incidence of MSD needs to be investigated with standardized and objective outcome measures to support and further establish the evidence found in this scoping review. A few biomechanical studies with a relatively small number of subjects provided useful information on the level of physical exposure during work by using objective measurements. These results should be confirmed in studies on larger samples under everyday conditions to derive valid recommendations for risk assessment and targeted preventive measures.

## Additional files

Additional file 1: **Table S1.** A Eligibility criteria; B Keywords included in the search strategy for all databases; terms searched for in the title and abstract of papers; C Database specific search strategies (DOCX 22 kb)

Additional file 2: **Table S2.** Summary of study characteristics (*N* = 44) (DOCX 31 kb)

## Data Availability

All data generated or analyzed during this study are included in this published article.

## Abbreviations

CI: Confidence interval
CTS: Carpal tunnel syndrome
EMG: Electromyography
ETD: Ergonomic tool design
MSD: Musculoskeletal disorders/discomfort
MVC: Maximal voluntary contraction
OR: Odds ratio
RR: Relative risk

## References

[1] Punnett L, Wegman DH (2004). Work-related musculoskeletal disorders: the epidemiologic evidence and the debate. J Electromyogr Kinesiol.

[2] Bernard BP. Musculoskeletal disorders and workplace factors - a critical review of epidemiologic evidence for work-related musculoskeletal disorders of the neck, upper extremity, and low back: National Institute of Occupational Safety and Health (NIOSH); 1997. http://www.cdc.gov/niosh/docs/97-141/pdfs/97-141.pdf. Accessed 05 June 2014

[3] Palmer KT (2011). Carpal tunnel syndrome: the role of occupational factors. Best Pract Res Clin Rheumatol.

[4] Silverstein B, Viikari-Juntura E, Kalat J (2002). Use of a prevention index to identify industries at high risk for work-related musculoskeletal disorders of the neck, back, and upper extremity in Washington state, 1990-1998. Am J Ind Med.

[5] Roquelaure Y, Ha C, Leclerc A, Touranchet A, Sauteron M, Melchior M (2006). Epidemiologic surveillance of upper-extremity musculoskeletal disorders in the working population. Arthritis Rheum.

[6] Kitzig D, Freitag S, Nienhaus A (2015). Musculoskeletal stress among hairdressers. Zbl Arbeitsmed.

[7] Veiersted KB, Gould KS, Osteras N, Hansson G-A (2008). Effect of an intervention addressing working technique on the biomechanical load of the neck and shoulders among hairdressers. Appl Ergon.

[8] Wahlström J, Mathiassen SE, Liv P, Hedlund P, Ahlgren C, Forsman M (2010). Upper arm postures and movements in female hairdressers across four full working days. Ann Occup Hyg.

[9] Miranda H, Viikari-Juntura E, Martikainen R, Takala EP, Riihimaki H (2001). A prospective study of work related factors and physical exercise as predictors of shoulder pain. Occup Environ Med.

[10] Svendsen SW, Bonde JP, Mathiassen SE, Stengaard-Pedersen K, Frich LH (2004). Work related shoulder disorders: quantitative exposure-response relations with reference to arm posture. Occup Environ Med.

[11] Chen HC, Chang CM, Liu YP, Chen CY (2010). Ergonomic risk factors for the wrists of hairdressers. Appl Ergon.

[12] Leino T, Kahkonen E, Saarinen L, Henriks-Eckerman ML, Paakkulainen H (1999). Working conditions and health in hairdressing salons. Appl Occup Environ Hyg.

[13] Bradshaw L, Harris-Roberts J, Bowen J, Rahman S, Fishwick D (2011). Self-reported work-related symptoms in hairdressers. Occup Med (Lond).

[14] Hassan OM, Bayomy H (2015). Occupational respiratory and musculoskeletal symptoms among Egyptian female hairdressers. J Community Health.

[15] Foss-Skiftesvik MH, Winther L, Johnsen CR, Zachariae C, Johansen JD (2017). Incidence of skin and respiratory diseases among Danish hairdressing apprentices. Contact Dermatitis.

[16] Leino T, Tuomi K, Paakkulainen H, Klockars M (1999). Health reasons for leaving the profession as determined among Finnish hairdressers in 1980-1995. Int Arch Occup Environ Health.

[17] Lysdal SH, Sosted H, Andersen KE, Johansen JD (2011). Hand eczema in hairdressers: a Danish register-based study of the prevalence of hand eczema and its career consequences. Contact Dermatitis.

[18] Arksey H, O’Malley L (2005). Scoping studies: towards a methodological framework. Int J Soc Res Methodol.

[19] Rumrill PD, Fitzgerald SM, Merchant WR (2010). Using scoping literature reviews as a means of understanding and interpreting existing literature. Work (Reading, Mass).

[20] Daudt HM, Mossel C, Scott SJ (2013). Enhancing the scoping study methodology: a large, inter-professional team’s experience with Arksey and O‘Malley’s framework. BMC Med Res Methodol.

[21] Agresti A, Coull BA (1998). Approximate is better than “exact” for interval estimation of binomial proportions. Am Stat.

[22] Diab KK, Nielsen J, Andersson E (2014). Swedish female hairdressers’ views on their work environment--a qualitative study. J Occup Health.

[23] Guo HR, Tanaka S, Cameron LL, Seligman PJ, Behrens VJ, Ger J (1995). Back pain among workers in the United States: national estimates and workers at high risk. Am J Ind Med.

[24] Roquelaure Y, Ha C, Nicolas G, Pelier-Cady MC, Mariot C, Descatha A (2008). Attributable risk of carpal tunnel syndrome according to industry and occupation in a general population. Arthritis Rheum.

[25] Schneider S, Lipinski S, Schiltenwolf M (2006). Occupations associated with a high risk of self-reported back pain: representative outcomes of a back pain prevalence study in the Federal Republic of Germany. Eur Spine J.

[26] Nanyan P, Ben CM. Compensation claims for work-related musculoskeletal disorders among hairdressers in France, 2010-2016. Int J Occup Saf Ergon. 2018:1–15. 10.1080/10803548.2018.1544743. 10.1080/10803548.2018.1544743 30412039

[27] Arokoski JP, Nevala-Puranen N, Danner R, Halonen M, Tikkanen R (1998). Occupationally oriented medical rehabilitation and Hairdressers’ work techniques--a one-and-a-half-year follow-up. Int J Occup Saf Ergon.

[28] Arokoski JPA, Juntunen M, Luikku J (2002). Use of health-care services, work absenteeism, leisure-time physical activity, musculoskeletal symptoms and physical performance after vocationally oriented medical rehabilitation-description of the courses and a one-and-a-half-year follow-up study with farmers, loggers, police officers and hairdressers. Int J Rehabil Res.

[29] Bertozzi L, Carpra F, Barducci C, Pillastrini P (2011). Effect of a physiotherapy program in the management of musculoskeletal disorders in hairdressers: a randomized controlled trial. It J Physiotherapy.

[30] Boyles JL, Yearout RD, Rys MJ (2003). Ergonomic scissors for hairdressing. Int J Ind Ergon.

[31] Crippa M, Torri D, Fogliata L, Belleri L, Alessio L (2007). Implementation of a health education programme in a sample of hairdressing trainees. Med Lav.

[32] Nevala-Puranen N, Halonen M, Tikkanen R, Arokoski J (1998). Changes in hairdressers’ work techniques and physical capacity during rehabilitation. Occup Ergon.

[33] Kitzig D, Hoehne-Hückstädt U, Freitag S, Glitsch U, Schedlbauer G, Ellegast R (2017). Body postures andmovement in typical hairdressing work. Feasibility study onmeasurement-based analysis. Zbl Arbeitsmed.

[34] Hanvold TN, Waersted M, Mengshoel AM, Bjertness E, Stigum H, Twisk J (2013). The effect of work-related sustained trapezius muscle activity on the development of neck and shoulder pain among young adults. Scand J Work Environ Health.

[35] Hanvold TN, Waersted M, Mengshoel AM, Bjertness E, Twisk J, Veiersted KB (2014). A longitudinal study on risk factors for neck and shoulder pain among young adults in the transition from technical school to working life. Scand J Work Environ Health.

[36] Hanvold TN, Waersted M, Mengshoel AM, Bjertness E, Veiersted KB (2015). Work with prolonged arm elevation as a risk factor for shoulder pain: a longitudinal study among young adults. Appl Ergon.

[37] Kaushik A, Patra P (2014). Upper extremity and neck disability in male hairdressers with concurrent changes in pinch strength: an observational study. Healthline.

[38] Tsigonia A, Tanagra D, Linos A, Merekoulias G, Alexopoulos EC (2009). Musculoskeletal disorders among cosmetologists. Int J Environ Res Public Health.

[39] Adewumi-Gunn TA, Ponce E, Flint N, Robbins W (2016). A preliminary community-based occupational health survey of black hair salon workers in South Los Angeles. J Immigr Minor Health.

[40] Amodeo M, Boudot H, Desfray F, Ducrot - Henry L, Gomis C, Seneque B (2004). La coiffure: une enquête de terrain en Côte-d’Or. Doc Méd Trav.

[41] Aweto HA, Tella BA, Johnson OY (2015). Prevalence of work-related musculoskeletal disorders among hairdressers. Int J Occup Med Environ Health.

[42] Cruz J, Dias-Teixeira M, Arezes PM, Santos Baptista J, Barroso MP, Carneiro P, Cordeiro P, Costa N, Melo RB, Miguel AS, Perestrelo G (2015). Prevalence of skeletal muscle injuries in hairdressers in the district of Setubal. Occupational safety and hygiene III.

[43] De Smet E, Germeys F, De Smet L (2009). Prevalence of work related upper limb disorders in hairdressers: a cross sectional study on the influence of working conditions and psychological, ergonomic and physical factors. Work (Reading, Mass).

[44] Deschamps F, Langrand J, Lesage F-X (2014). Health assessment of self-employed hairdressers in France. J Occup Health.

[45] Douwes M, Blatter BM, Eikhout SM, Bronkhorst RE, Michel FP. Osinga DSC. Onderzoek in het kader van het arboconvenant fysieke belasting bij kappers. TNO Arbeid. 2001; https://publications.tno.nl/publication/.../douwes-2001-onderzoek.pdf. Accessed 18 Aug 2017.

[46] Mahdavi S, Mahdavi S, Safari M, Rashidi R, Dehghani T, Kosari M (2013). Evaluation of the risk of musculoskeletal disorders using rapid entire body assessment among hairdressers in Khorramabad, Iran, in 2014. JOHE.

[47] Mandiracioglu A, Kose S, Gozaydin A, Turken M, Kuzucu L (2009). Occupational health risks of barbers and coiffeurs in Izmir. Indian J Occup Environ Med.

[48] Mussi G, Gouveia N (2008). Prevalence of work-related musculoskeletal disorders in Brazilian hairdressers. Occup Med (Lond).

[49] Nordander C, Ohlsson K, Akesson I, Arvidsson I, Balogh I, Hansson G-A (2013). Exposure-response relationships in work-related musculoskeletal disorders in elbows and hands - a synthesis of group-level data on exposure and response obtained using uniform methods of data collection. Appl Ergon.

[50] O'Loughlin M. How healthy are hairdressers? An investigation of health problems of female, Western Australian hairdressers: Edith Cowan University; 2010. https://ro.ecu.edu.au/cgi/viewcontent.cgi?article=1142&context=theses. Accessed 15 Aug 2017

[51] Omokhodion FO, Balogun MO, Ola-Olorun FM (2009). Reported occupational hazards and illnesses among hairdressers in Ibadan. Southwest Nigeria West Afr J Med.

[52] Puckree T (2009). Musculoskeletal pain in hairdressers- a study in Durban. JCHS.

[53] Demiryurek BE, Aksoy GA (2018). Prevalence of carpal tunnel syndrome and its correlation with pain amongst female hairdressers. Int J Occup Med Environ Health.

[54] Mastrominico E, Breschi C, Fattori GC, Pini F, Carnevale F (2007). Biomechanical overcharge of the upper limbs in hairdressers: from the task analysis to the job/exposition matrix. G Ital Med Lav Ergon.

[55] Figueiredo da Rocha L, Simonelli AP (2012). The use of ergonomic job analysis as a tool for the occupational therapist in the study of the labor activity of hairdressers. Cadernos de Terapia Ocupacional.

[56] Kilbom A (1994). Repetitive work of the upper extremity: part I—guidelines for the practitioner. Int J Ind Ergon.

[57] van der Molen HF, Foresti C, Daams JG, Frings-Dresen MHW, Kuijer PPFM (2017). Work-related risk factors for specific shoulder disorders: a systematic review and meta-analysis. Occup Environ Med.

[58] van Rijn RM, Huisstede BMA, Koes BW, Burdorf A (2010). Associations between work-related factors and specific disorders of the shoulder – a systematic review of the literature. Scand J Work Environ Health.

[59] van der Windt DAWM, Thomas E, Pope DP, de Winter AF, Macfarlane GJ, Bouter LM (2000). Occupational risk factors for shoulder pain: a systematic review. Occup Environ Med.

[60] Gallagher S, Heberger JR (2013). Examining the interaction of force and repetition on musculoskeletal disorder risk: a systematic literature review. Hum Factors.

[61] Kozak A, Schedlbauer G, Wirth T, Euler U, Westermann C, Nienhaus A (2015). Association between work-related biomechanical risk factors and the occurrence of carpal tunnel syndrome: an overview of systematic reviews and a meta-analysis of current research. BMC Musculoskelet Disord.

[62] van Rijn RM, Huisstede BM, Koes BW, Burdorf A (2009). Associations between work-related factors and specific disorders at the elbow: a systematic literature review. Rheumatology.

[63] Aptel M, Aublet-Cuvelier A, Claude CJ (2002). Work-related musculoskeletal disorders of the upper limb. Joint Bone Spine.

