# Supplementary material for: Musculoskeletal health, work-related risk factors and preventive measures in hairdressing: a scoping review
Source: J Occup Med Toxicol. 2019 Aug 17;14:24. doi: 10.1186/s12995-019-0244-y (PMC6698044; doi:10.1186/s12995-019-0244-y)
Supplement: Supplementary file 1 — Table S1. A Eligibility criteria; B Keywords included in the search strategy for all databases; terms searched for in the title and abstract of papers; C Database specific search strategies (DOCX 22 kb) [file 12995_2019_244_MOESM1_ESM.docx]

**Supporting material 1**

**Table S1**-**A** Eligibility criteria

| **Inclusion criteria** | **Exclusion criteria** |
| --- | --- |
| Population |  |
| - hairdressers, who continue to work and those who already left the profession with provision of reasons for leaving; - trainees, apprentices, self-employed, employed; - professions^1^ which overlap with hairdressing such as cosmetologist, barber, beautician; - mean age greater than 16 years; | - unemployed study population |
| Intervention |  |
| - includes all interventions that aim to prevent or reduce work-related MSD; | - intervention related to non-work-related MSD; |
| Outcome |  |
| - work-related disorders and/or diseases (e.g. medically confirmed diagnoses); - disorders related to musculoskeletal system such as (recurrent) pain, discomfort, tingling, numbness, stiff joints, swelling or dull aches; - all body regions (e.g. neck, lower and upper back; shoulder, arm, hand, finger, hip, knee, feet); | - no separate results for the anticipated study population; - medical conditions not related to work |
| Exposure |  |
| - exposure categories for MSD known from the literature such as: work-related biomechanical, psychological, organizational or individual risk factors | - only provision of exposures other than work-related |
| Study design |  |
| - peer-review and non-peer-review publications - cohort, case-control, cross-sectional observational studies; - qualitative studies; - ergonomic studies (e.g. biomechanical or observational task and/or posture analysis); - studies where prevalence data for MSD can be extracted or calculated; | - editorials and letters; - commentaries; - conference papers; - policy statements or expert opinions; - case reports |
| Time frame |  |
| - studies published since the inception of each database until November 5, 2018; |  |
| Languages |  |
| - English, German, Dutch, French, Italian, Portuguese and Spanish; | - languages that are not or only insufficiently mastered in the working group |

^1^ Cosmetology is the work of beauty therapists, including hairdressing, facials or manicures that provide cosmetic treatment of skin, hair and nails. In the German classification for the vocational training to become a hairdresser according to the training framework (§4 para. 2 FriseurAusbV 2008), hairdressers have to learn the following skills: decorative cosmetics and manicure as well as skin cosmetics and make-up skills. With regard to the location of the beauty services provided, i.e. mainly the head, we took into account all professions that perform tasks on this particular body region.

**Table S1-B** Keywords included in the search strategy for all databases; terms searched for in the title and abstract of papers

| **Population** | **Outcome** |
| --- | --- |
| Hairdresser* | musculoskeletal diseases[MeSH Terms] |
| Coiffeur | musculoskeletal symptoms |
| Beautician* | musculoskeletal pain |
| Cosmetologist* | musculoskeletal disorders |
| Barbering[MeSH Terms]^1^ | neck pain[MeSH Terms] |
| Beauty Culture[MeSH]^2^ | back pain[MeSH Terms] |
|  | shoulder pain[MeSH Terms] |
|  | upper extremity[MeSH Terms] |
|  | upper limb |

^1^ *Barbering* (MeSH term; definition in Pubmed): The occupation concerned with the cutting and dressing of the hair of customers and, of men, the shaving and trimming of the beard and mustache (Random House Unabridged Dictionary, 2d ed).

^2^ *Beauty Culture* (MeSH term; definition in Pubmed): An industry that creates products and procedures designed to enhance physical appearance and aesthetic appeal.

**Table S1-C** Database specific search strategies

| **Category** | **Keywords and MeSH headings** |
| --- | --- |
| **1. PUBMED** |  |
| Outcome | (((((((((((musculoskeletal*) **OR** musculoskeletal symptoms) **OR** musculoskeletal pain) **OR** upper limb*) **OR** upper extremity*[MeSH Terms]) **OR** neck pain[MeSH Terms]) **OR** back pain[MeSH Terms]) **OR** shoulder pain[MeSH Terms]) **OR** musculoskeletal diseases[MeSH Terms]) **OR** musculoskeletal disorders)) |
|  | **AND** |
| Population | (((((hairdress*) **OR** barbering*[MeSH Terms]) **OR** cosmetologist*) **OR** beautician*) **OR** coiffeur*) **OR** beauty culture*[MeSH Terms] |
| **2. LIVIVO** |  |
| Outcome | MESH=( ( ( ( ( ( ( ( ( ( musculoskeletal* ) **OR** musculoskeletal pain ) **OR** musculoskeletal disorders ) **OR** musculoskeletal symptoms ) **OR** upper limb* ) **OR** upper extremity*) **OR** neck pain) **OR** back pain) **OR** shoulder pain) **OR** musculoskeletal diseases) |
|  | **AND** |
| Population | ( ( ( ( ( ( hairdress* ) **OR** MESH=barbering* ) **OR** cosmetologist* ) **OR** beautician* ) **OR** coiffeur* ) **OR** MESH=(beauty culture) ) |
| **3. WEB of SCIENCE** |  |
| Outcome | (musculoskeletal*) **OR** (shoulder pain) **OR** (back pain) **OR** (neck pain) **OR** (musculoskeletal symptoms) **OR** (musculoskeletal disorders) **OR** (musculoskeletal pain) **OR** (upper limb*) **OR** (upper extremity*) |
|  | **AND** |
| Population | (hairdress*) **OR** (cosmetologist*) **OR** (barber*) **OR** (coiffeur*) |
| **4. MEDLINE (via OVID)** |  |
| Outcome | musculoskeletal*.mp. **OR** Musculoskeletal Diseases/ or musculoskeletal symptom*.mp. **OR** Musculoskeletal Diseases/ or musculoskeletal disorders.mp. **OR** musculoskeletal pain.mp. or Musculoskeletal Pain/ **OR** Shoulder Pain/ or Back Pain/ **OR** neck pain.mp. or Neck Pain/ **OR** upper limb*.mp. **OR** Cumulative Trauma Disorders/ or upper extremity.mp. or Upper Extremity/ or Nerve Compression Syndromes/ |
|  | **AND** |
| Population | hairdress*.mp. **OR** coiffeur*.mp. **OR** beauty culture.mp. or Beauty Culture/ **OR** barbering.mp. or Barbering/ **OR** cosmetologist*.mp **OR** beautician*.mp. |
| **5. CINAHL** |  |
| Outcome | musculoskeletal disorders **OR** musculoskeletal pain **OR** musculoskeletal diseases **OR** musculoskeletal symptoms **OR** upper limb disorders **OR** upper extremity or upper limb or hand or arm **OR** neck pain **OR** back pain **OR** shoulder pain |
|  | **AND** |
| Population | hairdress* **OR** barber* **OR** cosmetologist* **OR** beautician* |

*mp*=title, abstract, original title, name of substance word, subject heading word, keyword heading word, protocol supplementary concept word, rare disease supplementary concept word, unique identifier.
