# Supplementary material for: Musculoskeletal health, work-related risk factors and preventive measures in hairdressing: a scoping review
Source: J Occup Med Toxicol. 2019 Aug 17;14:24. doi: 10.1186/s12995-019-0244-y (PMC6698044; doi:10.1186/s12995-019-0244-y)
Supplement: Supplementary file 2 — Table S2. Summary of study characteristics (N = 44) (DOCX 31 kb) [file 12995_2019_244_MOESM2_ESM.docx]

**Supporting material 2**

**Table S2** Summary of study characteristics (N=44)

| **#** | **Author (year)** | **Study place** | **Design;**  **publication type** | **Population** | **N hair-dressers**  **(females)** | **Methodology** | **Measurements**  **(outcome or/and exposure)** | **Study question** |
| --- | --- | --- | --- | --- | --- | --- | --- | --- |
| 1 | Adewumi-Gunn et al. (2016) [39] | USA | cross-sectional;  peer review | black hair care workers | 22 (18 females) | questionnaire-based study with face to face interviews | (1) MSD (point prevalence) in different body sites | prevalence |
| 2 | Amodeo et al. (2004) [40] | France | cross-sectional;  non-peer review | hairdressers | 389 (not specified) | questionnaire-based study during annual visits to the occupational health service | (1) MSD (12-month prevalence);  (2) MSD severity (impeded work) in different body sites | prevalence |
| 3 | Arokoski et al. (1998) [27] | Finland | evaluation (pre-post);  peer review | hairdressers with chronic MS pain | 21 (all females) | evaluation of rehabilitation course (1.5-years follow-up) | (1) neck and back pain;  (2) work-related strains;  (3) changes in work techniques | rehabilitative measures |
| 4 | Arokoski et al. (2002) [28] | Finland | evaluation (pre-post);  peer review | hairdressers, loggers, police, farmers with MSD | 61 (all females) | evaluation of VOMR® rehabilitation course  (1.5-years follow-up)  – group comparisons | (1) neck and back pain;  (2) physical and mental strain;  (3) use of health-care services;  (4) work absenteeism;  (5) physical activity/performance | rehabilitative measures |
| 5 | Aweto et al. (2015) [41] | Nigeria | cross-sectional;  peer review | hairdressers | 299 (242 females) | questionnaire-based study during annually visits to the occupational health service | (1) MSD in different body sites using NQ (12-month prevalence) | prevalence; risk factors; strategies |
| 6 | Bertozzi et al. (2011) [29] | Italy | evaluation (pre-post);  peer review | hairdressers | 28 (all female) | evaluation of a 6-week exercise program for the lumbar and cervical spine in addition to an ergonomic brochure | (1) neck pain and LBP by using VAS;  (2) perceived level of disability as a result of MSD using the RMDQ and ODI Index; | preventive measures |
| 7 | Boyles et al. (2003) [30] | USA | Evaluation (pre-post);  peer review | hairdressers | 44 (41 female) | evaluation of new ETD scissors (bend in the handles of 90°) in comparison to standard scissors | (1) grip strength;  (2) perceived pain;  (3) frequency of wrist in bent or neutral position or above shoulder;  (4) usability | ergonomic tool design |
| 8 | Bradshaw et al. (2011) [13] | England | case-control;  peer review | hairdressers | 147 (all female) | questionnaire-based study  – group comparisons | (1) MSD in different body sites using the NQ (3-month prevalence) | prevalence; barriers |
| 9 | Chen et al. (2010) [11] | Taiwan | measurement study;  peer review | hairdressers/ barbers | 21 (10 females) | measurement study of upper extremities  – group comparisons | (1) wrist angles;  (2) forearm extensor and flexor;  (3) velocity and repetitiveness | postures/ movements |
| 10 | Crippa et al. (2007) [31] | Italy | evaluation (pre-post);  peer review | hairdressing trainees | 154 (144 female) | questionnaire-based study at the start and at the end of the school training (3 years later) | (1) health complaints;  (2) preventive measures;  (3) change in work activities;  (4) knowledge of occupational risks | preventive measures |
| 11 | Cruz & Dias-Teixeira (2015) [42] | Portugal | cross-sectional;  unknown | hairdressers | 30 (not specified) | questionnaire-based study | (1) MSD in different body sites (point prevalence);  (2) MSD duration, intensity, onset;  (3) subjectively assumed risk factors | prevalence; risk factors; |
| 12 | De Smet et al. (2009) [43] | Belgium | cross-sectional;  peer review | hairdressers | 145 (119 females) | questionnaire-based study | (1) WRULD intensity (pain during the activity >1 day or chronic pain);  (2) gripping force | prevalence;  risk factors; |
| 13 | Demiryurek & Gündogdu, 2017 [53] | Turkey | case-control;  peer review | hairdressers | 70 (all females) | measurement and questionnaire-based study of hairdressers and matched controls  – group comparisons | CTS measurements:  (1) Electroneuromyography (ENMG);  (2) Boston CTS Questionnaire;  (3) VAS | prevalence CTS |
| 14 | Deschamps et al. (2014) [44] | France | cross-sectional;  peer review | self-employed (SE) vs. wage earning (WE) hairdressers | 311 (275 females; SE=199;  WE=112) | questionnaire-based study during occupational health examination – group comparisons | (1) MSD related to repetitive movements (point prevalence);  (2) work-related stress | prevalence |
| 15 | Diab et al. (2014) [22] | Sweden | cross-sectional (qualitative);  peer review | hairdressers | 14 (all females) | qualitative study with face to face open ended interviews | (1) physical,  (2) social,  (3) psychological work environment | strategies & barriers |
| 16 | Douwes et al. (2001) [45] | Netherlands | cross-sectional;  non-peer review | hairdressers; screen workers | 280 (267 females) | questionnaire-based study  – group comparisons | (1) Repetitive Strain Injury (RSI);  (2) work-related risk factors;  (3) tasks and aids available | prevalence;  risk factors |
| 17 | Figueiredo da Rocha et al. (2012) [55] | Brazil | cross-sectional;  peer review | hairdressers/beauticians | 50 (tasks analysis n=4) | ergonomic job analysis of ergonomic risks of the working situation | (1) postures and movements during work;  (2) other occupational exposures | postures/ movements; |
| 18 | Foss-Skiftesvik et al. 2017 [15] | Denmark | cohort;  peer-review | hairdressing apprentices | 248 (239 females) | questionnaire-based longitudinal-study  (3-years follow-up)  – group comparisons | (1) discontinuation of hairdressing;  (2) health symptoms (e.g. NOSQ) and occupational exposures | leaving job |
| 19 | Guo et al. (1995) [23] | USA | surveillance data analysis;  peer review | multiple jobs (hairdressers/cosmetologist | not specified  N total  5,256 | data analysis of occupation-specific data from National Health Interview Survey (NHIS), based on Bureau of the Census occupational codes  – group comparisons | (1) LBP lasting over a week or more (population-based 12-month prevalence ratios) | prevalence |
| 20 | Hanvold et al. (2013) [34] | Norway | cohort;  peer review | hairdressers;  electricians; media/ design trainees | 15 (all females) | measurement- and questionnaire-based longitudinal study (2.5-years follow-up)  – group comparisons | (1) shoulder pain during the preceding 4 weeks;  (2) upper-trapezius muscle activity by using EMGmax | risk factors; postures/ movements; |
| 21 | Hanvold et al. (2014) [35] | Norway | cohort;  peer review | hairdressers;  electricians; media/ design trainees | 167 (163 females) | measurement- and questionnaire-based longitudinal study (6.5-years follow-up)  – group comparisons | (1) neck and shoulder pain during the < 4 weeks | risk factors; postures/ movements |
| 22 | Hanvold et al. (2015) [36] | Norway | cohort;  peer review | hairdressers  electricians; media/ design trainees | 15 (all females) | measurement- and questionnaire-based longitudinal-study (2.5-years follow-up)  – group comparisons | (1) shoulder pain during the <4 weeks;  (2) work with elevated arms by using inclinometers | risk factors; postures/ movements; |
| 23 | Hassan & Bayomy (2015) [14] | Egypt | case-control;  peer review | hairdressers; office workers | 80 (all females) | questionnaire-based study of hairdressers and matched controls  – group comparisons | (1) WRMSD in different body sites using the NQ (12-month prevalence);  (2) chronic pain (≥3 months);  (3) visits to the doctor or sickness absence due to WRMSD | prevalence; risk factors |
| 24 | Kaushik & Patra (2014) [37] | India | cross-sectional;  unknown | hairdressers | 59 (all males) | questionnaire-based study | 1) MSD using the NPDI and DASH index;  (2) measurement of pinch strength | risk factors |
| 25 | Kitzig et al. (2015) [6] | Germany | cross-sectional;  peer review | hairdressers | 5 (all females) | (1) analysis of routine data from health insurance companies;  (2) analysis of data from accident insurance;  (3) observational study of video recordings of five hairdressers | (1) sick leave;  (2) occupational diseases;  (3) ergonomic postures during frequent tasks | postures/ movements |
| 26 | Kitzig et al. (2017) [33] | Germany | measurement study;  peer review | hairdresser | 1 female | measurement study of postures and movements during work by using the CUELA system | (1) body postures and movements | postures/ movements |
| 27 | Leino et al. (1999) [12] | Finland | cross-sectional;  peer review | hairdressers | 85 (not specified) | questionnaire-based study and assessment of physical and chemical work environment | (1) work factors most hazardous to health or caused a disease;  (2) MSD diagnosis by physician | risk factors |
| 28 | Leino et al. (1999) [16] | Finland | case-control; peer review | hairdressers; commercial work | 3484 (all females) | questionnaire-based study on hairdressers and controls  – group comparisons | (1) reasons for leaving the hairdressing trade within 15 years of follow-up (1980-1995) | leaving job |
| 29 | Lysdal et al. 2011 [17] | Denmark | cross-sectional;  peer-review | hairdressing graduates | 5239 (5015 females) | Register-based questionnaire study of graduates from 1985-2007 | (1) reasons for leaving the hairdressing trade;  (2) health symptoms (e.g. NOSQ) and occupational exposures | leaving job |
| 30 | Mahdavi et al. (2013) [46] | Iran | cross-sectional; peer review | hairdressers | 172 (all females) | questionnaire-based study and task analysis | (1) MSD using the NQ (no time frame);  (2) ergonomic analysis using REBA | prevalence; risk factors |
| 31 | Mandiracioglu et al. (2009) [47] | Turkey | cross-sectional; unknown | hairdressers, barbers | 1284 (not specified) | questionnaire-based study after training in occupational health | (1) MS discomfort (12-month prevalence) | prevalence |
| 32 | Mastro-minico et al. (2007) [54] | Italy | cross-sectional;  unknown | hairdressers | 12 (7 females) | observational study of hairdressing tasks by using the OCRA check list | (1) OCRA index (score >4.6 risk for ULD) | risk factors |
| 33 | Mussi & Gouveia (2008) [48] | Brazil | cross-sectional; peer review | hairdressers | 220 (not specified) | questionnaire-based study | (1) MSD using the NQ (lasted > 6 months with a frequency of at least once a month) | prevalence; risk factors |
| 34 | Nanyan & Charrada, 2018 [26] | France | register data analysis;  peer-review | hairdressers | >90% females | register-based data of compensation claims for WRMSDs from the French National Health Insurance Fund | (1) number of claims (WRMSD)  (2) permanent disability  (2) lost work days | compensation claims |
| 35 | Nevala-Puranen et al. (1998) [32] | Finland | evaluation (pre-post);  peer review | hairdressers with history of MSD | 10 (all females) | evaluation of rehabilitation course of hairdressers on sick leave due to MSD for max. 60 days (1.5-years follow-up) | (1) muscle activity (%MVC);  (2) physical capacity (VO_2_max);  (3) muscle strength/endurance;  (4) MS pain intensity;  (5) perceived work ability;  (6) workspace redesign | rehabilitative measures |
| 36 | Nordander et al. (2013) [49] | Sweden | cross-sectional;  peer review | multiple jobs (hairdressers) | 78 (all females) | questionnaire- and measurement-based study of 27 occupations  – group comparisons | (1) MSD using the NQ (12-month and 7-day prevalence);  (2) wrist postures and velocity;  (3) muscular load;  (4) psychosocial exposure | prevalence; postures/ movements; |
| 37 | Omokhodion et al. (2009) [51] | Nigeria | cross-sectional;  peer review | hairdressers (i.a. trainees) | 355 (all females; | questionnaire-based study with face to face interviews | (1) self-reported illnesses;  (2) occupational accidents | prevalence |
| 38 | O'Loughlin (2010) [50] | Australia | cross-sectional;  non-peer review | hairdressers | 238 (all females) | questionnaire-based study | (1) MSD and other health problems (12-month prevalence) | prevalence |
| 39 | Puckree (2009) [52] | South Africa | cross-sectional;  peer review | hairdressers | 75 (all females) | questionnaire-based study | (1) MSD (point prevalence);  (2) pain intensity;  (3) arm posture, bending | risk factors |
| 40 | Roquelaure et al. 2008 [24] | France | surveillance data analysis;  peer-review | multiple jobs  (hairdressers) | not specified | epidemiologic surveillance data from Maine and Loire regions for the years 2002 to 2004 | (1) attributable risk fractions of CTS among exposed persons | incidence CTS |
| 41 | Schneider et al. (2006) [25] | Germany | surveillance data analysis;  peer review | multiple jobs (hairdressers/beauticians) | 26 (not specified) | analysis of occupation-specific data from First National Health Survey – group comparisons | (1) LBP (7-day and 12-month prevalence) | prevalence |
| 42 | Tsigonia et al. (2009) [38] | Greece | cross-sectional;  peer review | cosmetologist | 102 (95 females) | questionnaire-based study | (1) MSD in neck and shoulder using the NQ (12-month prevalence);  (2) health status;  (3) physical & psychosocial exposure; | prevalence; risk factors |
| 43 | Veiersted et al. (2008) [7] | Norway | evaluation (pre-post); peer review | hairdressers | 188 (all females;  38 in the intervention group) | (1) questionnaire and measurement study;  (2) evaluation of working instructions  (follow-up of 2 months) | (1) MSD in neck and shoulder using the NQ (12-month prevalence);  (2) arm elevation using inclinometer;  (3) muscular load of m. trapezius | prevalence; preventive measures;  postures/ movements |
| 44 | Wahlström et al. (2010) [8] | Sweden | measurement study;  peer review | hairdressers | 28 (all females) | ergonomic measurement study of upper arm postures and movements | (1) upper arm postures  (2) customer and non-customer tasks | postures/ movements |

*Abbreviations:* *DASH* Disability of Arm, Shoulder, Hand Index; *CTS* Carpal Tunnel Syndrome; *CUELA* Computer-Assisted Recording and Long-term Analysis of Musculoskeletal Loads; *EMGmax* Maximum Muscle Contractions; *ETD* Ergonomic Tool Design; *LBP* Low back pain; *MS* musculoskeletal; *MSD* Musculoskeletal Disorders; *NPDI* Neck Pain Disability Index; *NQ* Nordic Questionnaire; *NOSQ* Nordic Occupational Skin Questionnaire; *OCRA* Occupational Repetitive Action check list; *ODI* Oswestry Disability Index; *REBA* Rapid Entire Body Assessment; *RMDQ* Roland Morris Disability Questionnaire; *ULD* Upper Limb Disorders; *VAS* Visual Analogue Scale; *VO_2_max* maximum oxygen intake in milliliters; *WRMSD* Work-Related Musculoskeletal Disorders; *WRULD* Work-Related Upper Limb Disorders; *%MVC* Maximum Voluntary Contraction in %.
